# Catalogue of Medical, Surgical, Dental, and Scientific Books

**Published:** 1863-08

**Authors:** 


CATALOGUE OF MEDICAL, SURGICAL, DENTAL, AND SCIENTIFIC BOOKS. Published and for sale by LINDSAY & BLAKISTON, No. 25 South Sixth Street, Philadelphia.

This is a full catalogue of valuable medical and scientific books, with the publishers' prices attached. Those wishing to add to their libraries would do well to consult this catalogue.

